# Author Correction: Over-reliance on land for carbon dioxide removal in net-zero climate pledges

**DOI:** 10.1038/s41467-024-54087-3

**Published:** 2024-11-06

**Authors:** Kate Dooley, Kirstine Lund Christiansen, Jens Friis Lund, Wim Carton, Alister Self

**Affiliations:** 1 School of Geography, Earth and Atmospheric Sciences, The University of Melbourne, Parkville, VIC Australia; 2 Department of Food and Resource Economics, University of Copenhagen, Frederiksberg C, Denmark; 3 Centre for Sustainability Studies, Lund University, Lund, Sweden; 4 Climate Resource, Melbourne, VIC Australia

**Keywords:** Climate-change mitigation, Climate-change mitigation, Climate-change policy, Geography

Correction to: *Nature Communications* 10.1038/s41467-024-53466-0, published online 23 October 2024

The original version of this Article contained an error in the displayed percentages in Fig. 4. The correct version includes Fig. 4 with the corrected values. This has been corrected in both the PDF and HTML versions of the Article.

