# Author Correction: Over-reliance on land for carbon dioxide removal in net-zero climate pledges

**DOI:** 10.1038/s41467-025-58599-4

**Published:** 2025-04-09

**Authors:** Kate Dooley, Kirstine Lund Christiansen, Jens Friis Lund, Wim Carton, Alister Self

**Affiliations:** 1 School of Geography, Earth and Atmospheric Sciences, The University of Melbourne, Parkville, VIC Australia; 2 Department of Food and Resource Economics, University of Copenhagen, Frederiksberg C, Denmark; 3 Centre for Sustainability Studies, Lund University, Lund, Sweden; 4 Climate Resource, Melbourne, VIC Australia

**Keywords:** Climate-change mitigation, Climate-change mitigation, Climate-change policy, Geography

Correction to: *Nature Communications* 10.1038/s41467-024-53466-0, published online 23 October 2024

In the version of the article initially published, there was an error in Fig. 1, where the “Bioenergy” area was not embedded within the “Unconditional pledges” section. This has now been corrected in the HTML and PDF versions of the article, as seen in Fig. 1. Additionally, the Source data has also been amended to correct the same error.

Fig. 1 Original:
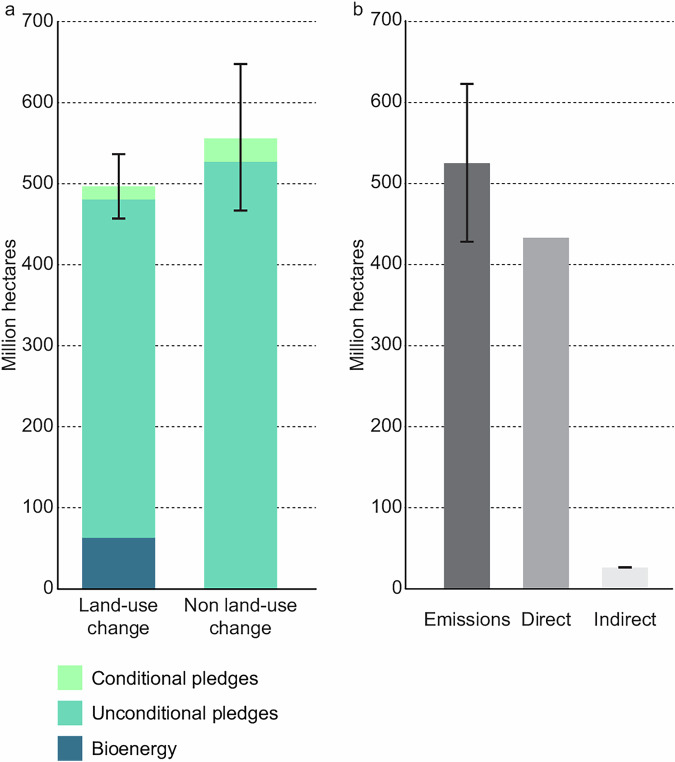


Fig. 1 Corrected:
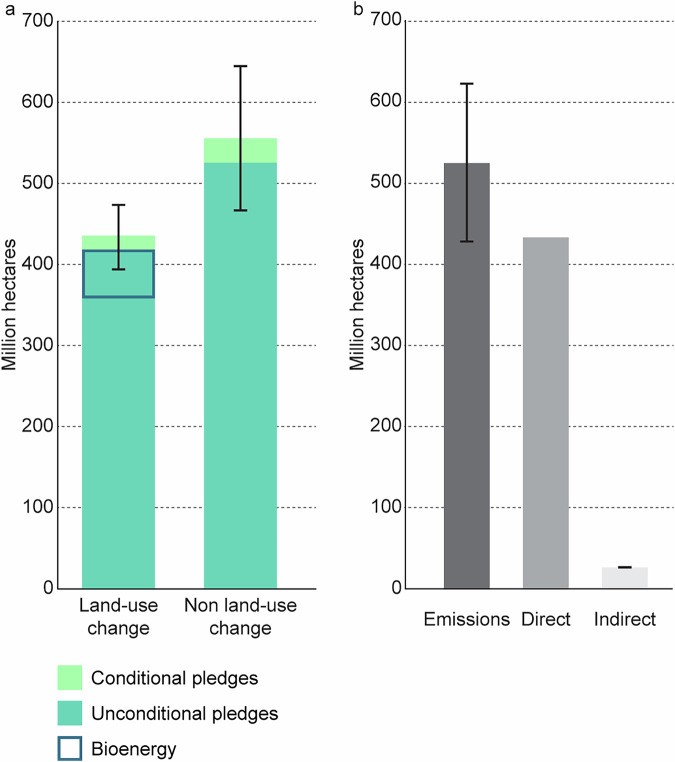


## Source data

Updated Source data

