# Supplementary material for: Over-reliance on land for carbon dioxide removal in net-zero climate pledges
Source: Nat Commun. 2024 Oct 23;15:9118. doi: 10.1038/s41467-024-53466-0 (PMC11496795; doi:10.1038/s41467-024-53466-0)
Supplement: Supplementary file 1 — Supplementary Information [file 41467_2024_53466_MOESM1_ESM.pdf]

Supplementary Information

Over-reliance on land for carbon dioxide removal in net-zero climate pledges

Kate Dooley[1], Kirstine Lund Christiansen*[2], Jens Friis Lund[2], Wim Carton[3], Alister Self[1,4].

[1] School of Geography, Earth and Atmospheric Sciences, The University of Melbourne, Parkville 3010, Australia.
[2] Department of Food and Resource Economics, University of Copenhagen, Rolighedsvej 23, 1958 Frederiksberg C, Denmark.
[3] Centre for Sustainability Studies, Lund University, Biskopsgatan 5, 223 62 Lund, Sweden.
[4] Climate Resource, Melbourne, 3065, Australia.
* Corresponding author: <klc@ifro.ku.dk>

Discussion

Uncertainty analysis

Land required, unless directly stated by the country in its climate pledge, was calculated by one of three methods depending on the pledge type: removal factors when pledges were made as tonnes CO_2 removed; tree density per ha when pledges were made as an increase in number of trees; and proportion of forest or land area when a proportional increase in forest area was pledged. We classified these as "Emissions pledges" where tonnes CO_2 removed were given, and "Indirect pledges" for those made as number of trees planted or proportion of forests / land area increase (see online methods). When countries directly stated land area required, these were classified as "Direct pledges".

Each of these approaches required different denominators and different approaches to estimate uncertainty. Removal Factors (RF) were calculated at the level of climate domain and activity type. For natural forests (Old Secondary, Young Secondary), variances and SD were sourced from Harris et al 2021 and Gibbs & Harris 2021. No variance was available for Plantations, which was imputed from Young Secondary as having the nearest RF. 95% confidence intervals and corresponding sample sizes were available for Agroforestry, Silvopasture, and Mangroves (Table S1). Assuming that these intervals were based on Student's *t* distributions, the corresponding sample SDs were computed (see Table S1).

The yield uptake rates and SD for bioenergy were taken from Li et al 2020. To convert gridded data in Li et al. to country specific yields we applied country specific polygons in QGIS, extracting the value from each pixel for 'best crop estimate' and then calculating the median per country. This method resulted in a global median of 16.12 compared to 16.4 reported by Li et al 2020. Conversion efficiency of 60% was applied to yield uptake and SD following Vaughan et al 2018. We note that the key process and land use change emissions that can influence the net CO_2 removed by a BECCS system is not easily quantified in a single value, such as conversion efficiency, and this is treated differently across different approaches to quantifying BECCS uptake (Vaughan et al 2018), with potentially large differences in results.

The SD for tree density per hectare was provided by Crowther et al 2015 (Table S2). We calculated the SD assuming a normal distribution. Forest and land areas per country were sourced from FAOSTAT 2023. These values are given as estimates, with no calculation for uncertainties.

Sensitivity analysis
We conducted a sensitivity analysis using a global average removal factor for all pledges. This removed any interpretative assumptions related to activity type or location. This resulted in a 8.4% increase in land area, showing that the use of biome and activity specific removal factors constrains the calculation of land area, but selection of activity types does not significantly drive the land area calculations. This is due to just under half of the results being based on direct or indirect pledges, as well as removal factor values falling within a relatively limited range.

Removal factors are used for above-ground biomass only (AGB). We carried out a sensitivity analysis, adding the below ground biomass (BGB) increment to all activities except forests remaining forests, in line with IPCC Tier 1 guidance (no net change to non-biomass pools). AGB + BGB removal factors are applied for mangroves, plantations, young secondary forests, silvopasture and agroforestry (Table S3). This resulted in a decrease in total land area of 8.9 million ha, which represents <1% of results. Below-ground biomass increments do not significantly impact the results given the relevant activity categories from emissions-based pledge cover only 36 million ha (see Supplementary data, tab Sens_BGB). Dead organic matter and soil carbon pools are not included (including for bioenergy crops) due to significant uncertainty around fluxes in these carbon pools.

Table S1. Removal Factors

**Climate domain**	**Activity type**	**AGB RF (Mg CO2 ha-1 yr-1)**	**s.d.**	**Source**
Boreal	Old Secondary	1.9	0.23	Harris et al 2021<br>Gibbs & Harris 2021
Boreal	Young Secondary	3.69	0.14	Harris et al 2021<br>Gibbs & Harris 2021
Boreal	Plantation	10.5	0.14	Harris et al 2021<br>*(s.d. imputed from Young Secondary)*
Subtropical	Old Secondary	2.68	0.44	Harris et al 2021<br>Gibbs & Harris 2021
Subtropical	Young Secondary	7.77	1.32	Harris et al 2021<br>Gibbs & Harris 2021
Subtropical	Plantation	13.2	1.32	Harris et al 2021<br>*(s.d. imputed from Young Secondary)*
Subtropical	Agroforestry	6.66	9.99	IPCC (RF calculated as average of Tropical RFs in Table 5.1)
Subtropical	Silvopasture	10.67	3.31	IPCC (RF calculated as for Tropical in Table 5.1)
Subtropical	Mangroves	33.03	1.15	IPCC 2013 (Table 4.4)
Tropical	Old Secondary	3.55	1.01	Harris et al 2021<br>Gibbs & Harris 2021
Tropical	Young Secondary	12.3	1.99	Harris et al 2021<br>Gibbs & Harris 2021
Tropical	Plantation	16.7	1.99	Harris et al 2021<br>*(s.d. imputed from Young Secondary)*
Tropical	Agroforestry	6.66	9.99	IPCC (RF calculated as average of Tropical RFs in Table 5.1)
Tropical	Silvopasture	10.67	3.41	IPCC (RF calculated as average of Tropical RFs in Table 5.1)
Tropical	Mangroves	12.05	2.40	IPCC 2013 (Table 4.4)
Temperate	Old Secondary	6.19	21.84	Harris et al 2021

				Gibbs & Harris 2021
Temperate	Young Secondary	4.83	0.30	Harris et al 2021<br>Gibbs & Harris 2021
Temperate	Plantation	11.1	0.30	Harris et al 2021<br>*(s.d. imputed from Young Secondary)*
Temperate	Agroforestry	3.19	0.85	IPCC 2019 (Table 5.1)
Temperate	Silvopasture	8.55	2.30	IPCC 2019 (Table 5.1)
Australia	Bioenergy	16.65	3.41	Li et al (2020) (best bioenergy crop, 60% conversion efficiency)
Canada	Bioenergy	9.88	2.31	Li et al (2020) (best bioenergy crop, 60% conversion efficiency)
Switzerland	Bioenergy	14.16	3.11	Li et al (2020) (best bioenergy crop, 60% conversion efficiency)
United Kingdom	Bioenergy	17.31	2.55	Li et al (2020) (best bioenergy crop, 60% conversion efficiency)
United States of America	Bioenergy	13.26	4.79	Li et al (2020) (best bioenergy crop, 60% conversion efficiency)

Table S2. Tree density

**Climate domain / Ecoregion**	**Tree density per hectare**	**s.d.**	**Source**
Boreal Forests	749.3	50.1	Crowther et al 2015
Temperate broadleaf	362.6	2.9	Crowther et al 2015
Temperate grasslands	148.3	4.9	Crowther et al 2015
Tropical dry	156.4	63.4	Crowther et al 2015
Tropical moist	799.4	24	Crowther et al 2015
Deserts	53	2.9	Crowther et al 2015
Mediterranean forests	53.4	1.2	Crowther et al 2015

Table S3. Including belowground biomass increment for sensitivity analysis

**Climate domain**	**Activity type**	**AGB+BGB Removal Factor (Mg CO_2 /ha /yr)**	**Source**
Boreal	Young Secondary	4.65	Harris et al 2021
Boreal	Plantation	13.20	Harris et al 2021
Subtropical	Young Secondary	9.79	Harris et al 2021
Subtropical	Plantation	16.60	Harris et al 2021
Subtropical	Agroforestry	7.10	IPCC 2019 (RF calculated as average of Tropical agroforestry systems in Table 5.2)
Subtropical	Silvopasture	11.46	IPCC 2019 (RF calculated as for Tropical in Table 5.2)
Subtropical	Mangroves	58.50	Harris et al 2021
Tropical	Young Secondary	15.50	Harris et al 2021
Tropical	Plantation	21.00	Harris et al 2021
Tropical	Agroforestry	7.10	IPCC (RF calculated as average of agroforestry systems in Table 5.2)
Tropical	Silvopasture	11.46	IPCC (RF calculated as average of Tropical agroforestry systems in Table 5.2)
Tropical	Mangroves	22.90	Harris et al 2021
Temperate	Young Secondary	9.79	Harris et al 2021
Temperate	Plantation	14.00	Harris et al 2021
Temperate	Agroforestry	3.42	IPCC 2019 (Table 5.2)
Temperate	Silvopasture	9.25	IPCC 2019 (Table 5.2)

References

Crowther, T. W., H. B. Glick, K. R. Covey, C. Bettigole, D. S. Maynard, S. M. Thomas, J. R. Smith, et al. "Mapping Tree Density at a Global Scale." *Nature* 525, no. 7568 (September 10, 2015): 201–5. https://doi.org/10.1038/nature14967.

FAO. "FAOSTAT License: CC BY-NC-SA 3.0 IGO. (Forest and Agriculture Organization of the United Nations (FAO))." Accessed July 25, 2024. http://www.fao.org/faostat/en/#data/RL.

Gibbs, David, and Nancy Harris. "Forest Carbon Removal Factor Variance by Climate Domain (1.2.0) [Dataset]." Dataset, 2021. https://doi.org/10.5281/zenodo.5537134.

Harris, Nancy L., David A. Gibbs, Alessandro Baccini, Richard A. Birdsey, Sytze de Bruin, Mary Farina, Lola Fatoyinbo, et al. "Global Maps of Twenty-First Century Forest Carbon Fluxes." *Nature Climate Change* 11, no. 3 (March 2021): 234–40. https://doi.org/10.1038/s41558-020-00976-6.

IPCC. *2013 Supplement to the 2006 IPCC Guidelines for National Greenhouse Gas Inventories: Wetlands: Methodological Guidance on Lands with Wet and Drained Soils, and Constructed Wetlands for Wastewater Treatment*. Geneva, Switzerland: IPCC, Intergovernmental Panel on Climate Change, 2014.

IPCC. *2019 Refinement to the 2006 IPCC Guidelines for National Greenhouse Gas Inventories. Volume 4, Chapter 5. Cropland.* Geneva, Switzerland: IPCC, Intergovernmental Panel on Climate Change, 2019.

Li, Wei, Philippe Ciais, Elke Stehfest, Detlef Van Vuuren, Alexander Popp, Almut Arneth, Fulvio Di Fulvio, et al. "Mapping the Yields of Lignocellulosic Bioenergy Crops from Observations at the Global Scale." *Earth System Science Data* 12, no. 2 (April 2, 2020): 789–804. https://doi.org/10.5194/essd-12-789-2020.

Vaughan, Naomi E, Clair Gough, Sarah Mander, Emma W Littleton, Andrew Welfle, David E H J Gernaat, and Detlef P Van Vuuren. "Evaluating the Use of Biomass Energy with Carbon Capture and Storage in Low Emission Scenarios." *Environmental Research Letters* 13, no. 4 (April 1, 2018): 044014. https://doi.org/10.1088/1748-9326/aaaa02.
